# Determinants of dental caries in children in the Middle East and North Africa region: a systematic review based on literature published from 2000 to 2019

**DOI:** 10.1186/s12903-021-01482-7

**Published:** 2021-05-04

**Authors:** Amal Elamin, Malin Garemo, Anzelle Mulder

**Affiliations:** Department of Health Sciences, College of Natural and Health Sciences, Zayed University, P.O. Box 144534, Abu-Dhabi, United Arab Emirates

**Keywords:** Children, Dental caries, Eating habits, Middle East, Northern Africa, Oral health, Risk factors, Socioeconomics, Sugar intake, Tooth brushing

## Abstract

**Background:**

Dental caries risk factors have been expanded to not only emphasize biology, dietary and oral habits but also broader social determinants such as socioeconomic factors and the utilization of health services. The aim was to review sociobehavioural/cultural and socioeconomic determinants of dental caries in children residing in the Middle East and North Africa (MENA) region.

**Methods:**

A search was conducted in the PubMed/Medline database and Google Scholar to identify studies published from 2000 to 2019 covering children using key search terms. In the initial stages, titles, abstracts and, if needed, full articles were screened for eligibility. In the final stage, all included articles were reassessed and read, and relevant data were extracted.

**Results:**

Out of 600 initial articles, a total of 77 were included in this review, of which 74 were cross-sectional, 2 were longitudinal and one was a case–control study. The studies included a total of 94,491 participants in 14 countries across the MENA region. A majority used the World Health Organization scoring system to assess dental caries. The caries prevalence ranged between 17.2% and 88.8%, early childhood caries between 3% and 57% and decayed missing filled teeth (dmft) varied between 0.6 and 8.5 across the various age groups. Increased age, low maternal education, low overall socioeconomic status, decreased frequency of tooth brushing, low parental involvement, poor oral habits, infant feeding practices and sugar consumption were among the most prevalent determinants for increased risk of caries in the reviewed studies.

**Conclusions:**

Dental caries was found to be high among children in many of the studies published from MENA. The key determinants of dental caries were found to include factors related to child characteristics, family background, oral hygiene and infant feeding and eating habits. The high dental caries prevalence emphasises the need to address the prevailing modifiable sociobehavioural and socioeconomic determinants by translating them into effective oral health prevention policies and programmes.

## Background

Dental caries continues to be one of the most prevalent chronic diseases worldwide and a costly burden to healthcare services despite the availability of effective basic prevention measures [1]. Since the declaration of the Millennium Development Goals (MDGs) in 2000 and later the Sustainable Development Goals (SDGs), both of which allowed for tracking countries’ health profiles, the profile of the Middle East and North Africa (MENA) region has undergone notable changes [2]. In some MENA countries, political stability, economic growth and investments in healthcare systems have led to improvements in various health indicators, whereas some countries have been impacted by political unrest or war; subsequently, the region currently includes low-middle income, upper-middle income and high income countries [3, 4]. These societal changes have also contributed to an increased rate of non-communicable diseases and persistence of some communicable diseases, such as dental caries, due to a marked shift in lifestyle, increased food availability and a notable nutritional transition among citizens [4].

Globally, the profile of dental caries is also heterogeneous across developing and developed countries, with large disparities reported between and within groups [5, 6]. Principally, it has been claimed that dental caries is decreasing in most industrialized countries due to improvements in prevention programmes and increased access to dental health services, but conflicting results have shown that dental caries is still prevalent among underprivileged groups in many of these countries [5, 7, 8]. In most developing countries, dental caries levels were low until recent years, after which an increase has been observed due to growing consumption of sugars, inadequate exposure to fluorides and limited access to oral healthcare services [5, 8, 9]. In the MENA region, trends in dental caries have shown a rapid increase in the incidence of the disease, with most caries remaining untreated [10]. Existing data from the Eastern Mediterranean Region (EMRO) from 20 countries show wide variations in dental caries with decayed, missing, and filled teeth scores (DMFT) among 12-year-olds ranging from 0.4 to 4.4 and a higher prevalence and severity of dental caries in the primary dentition than in the permanent dentition among 6-year-olds [10]. Furthermore, distinctions between dental caries experiences are present, with high rates of untreated caries in developing countries, which reflects the limited resources available and difficulties in accessibility and affordability to essential oral health care services [10, 11].

While determinants that contribute to the initiation and progression of dental caries are complex and multifactorial, understanding their role is crucial for establishing appropriate prevention and management strategies [12]. The determinants can be divided into biological, contextual/environmental, sociobehavioural/cultural and socioeconomic factors [13, 14]. Examples of biological determinants include host susceptibility and oral flora, and the contextual/environmental determinants include access to and utilization of dental healthcare services, oral health promotion programmes and fluoridation of water [15]. Moreover, examples of sociobehavioural/cultural determinants regarding dental caries include dental hygiene practices, consumption of sugars, lifestyle habits such as alcohol consumption and tobacco use [16]. To the best of our knowledge, there are no recent studies focusing on sociobehavioural/cultural and socioeconomic determinants of dental caries in children residing in the MENA region. Hence, the aim of the review was to address this gap in the literature.

The central questions for this review, which incorporated literature from 2000 to 2019 published from the MENA region were:

1. What sociobehavioural and socioeconomic variables have been studied within the context of dental caries prevalence in children, aged 0–20 years?
2. What did the reviewed studies reveal about the influence of sociobehavioural and socioeconomic variables on the risk for dental caries in children?
3. What recommendations can be made for future research?

## Methods

Electronic searches of databases (PubMed and Medline) supplemented by the use of an online search engine (Google Scholar) were used to explore determinants and prevalence of early childhood caries (ECC) or dental caries in children and young adults (age 0–20 years) residing in the MENA region*.* The World Atlas categorization of the MENA region was used, and accordingly, the following countries were included: Algeria, Bahrain, Egypt, Iran, Iraq, Jordan, Kuwait, Lebanon, Libya, Morocco, Oman, Palestine, Qatar, Saudi Arabia, Syria, Tunisia, Turkey, UAE and Yemen. Combinations of the following MeSH terms were used to identify relevant articles: “caries”, “children”, “determinants”, “behaviours”, “dietary causes”, “dietary habits”, “education, factors, income, socio, social determinants and geographic context (each of the individual countries, e.g., Egypt, Middle East and North Africa). An example of the search strategy used to search MEDLINE: (“determinant” [all fields] AND “caries” [all fields] AND “children” [all fields] AND “country name” [all fields]). Table 1 describes the search terms and examples of search strategies.

**Table 1:** Search terms and examples of search strategies using PubMed, Medline and Google scholar

Search category	Search words
Children	Children
Dental caries	Caries
Determinants	Behaviours, Determinants, Dietary causes, Dietary habits, Education, Factors, Income, Socio, Social determinants
Geographic context^a^	Algeria, Bahrain, Egypt, Iran, Iraq, Jordan, Kuwait, Lebanon, Libya, Morocco, Oman, Palestine, Qatar, Saudi Arabia, Syria, Tunisia, Turkey, UAE, Yemen, Middle East, North Africa
Examples of search strategies	Determinants AND caries AND children AND Middle East Factors AND caries AND children AND North Africa Behaviours AND caries AND children AND Algeria Socio AND caries AND children AND Bahrain Dietary causes AND caries AND children AND Egypt Dietary habits AND caries AND children AND Iran Education AND caries AND children AND Iraq Income AND caries AND children and Jordan Social determinants AND caries AND children AND Kuwait

^a^Countries being part of the Middle East and North Africa (MENA) according to the World Atlas categorization, 2018

### Screening process

A comprehensive literature search was performed and updated until June 2020. One author (AM) undertook the literature search in the specified search databases after which the two other authors (AE and MG) removed all the duplicates, identifying 600 articles. The titles and abstracts of the 600 articles were read by all authors and screened for relevance. AE and MG applied the inclusion and exclusion criteria, and when in doubt about the eligibility of an article, both independently read the abstract and, if necessary, the full-text article, after which it was discussed and full consensus was reached.

Duplicate references were checked and removed using Endnote bibliographic software [17].

### Inclusion and exclusion criteria

**Fig. 1:**
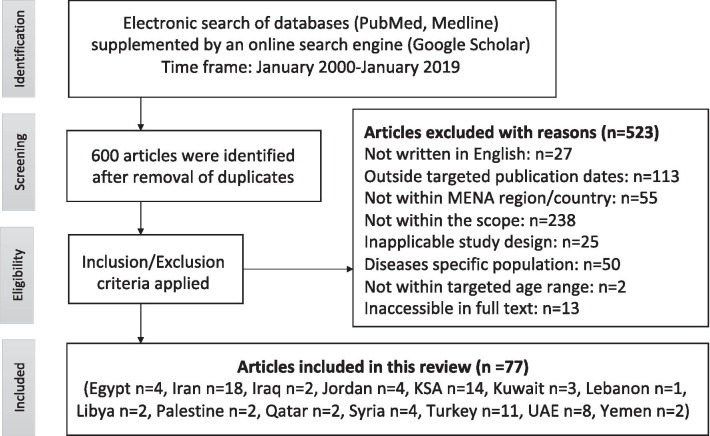
Flow chart of the literature search

From the identified 600 articles the inclusion and exclusion criteria were applied. The initial screening process was conducted to include only articles in English published during January 2000-January 2019 within the MENA region. Following this, the titles, abstracts and, when needed, the articles’ full text were screened according to their relevance to the scope of this study, the study design, health and medical conditions in the studied population and finally the age group. Articles that were not relevant to sociocultural, sociobehavioural and socioeconomic determinants of dental caries, such as those examining microbiological and genetic predictors of dental caries, were outside the scope of this study and were therefore excluded. Original cross-sectional studies, case–control studies and longitudinal studies were included, whereas reviews, interventional studies, case reports and editorial commentaries were excluded. Furthermore, studies focusing on children/young adults with certain health and medical conditions (cardiovascular disease, autism, diabetes, Down syndrome, etc.) were excluded. The final inclusion criterion that was applied was age; articles reporting results from children, teenagers and young adults aged 0–20 years were included, whereas findings related to adults were excluded. A few relevant articles where the full-text articles were not accessible were also excluded. This resulted in 77 articles being included for this study, and 523 articles were excluded as described in Fig. 1.

## Results

Overall, 77 articles were included in this review from 14 countries: Egypt (n = 4) [18–21], Iran (n = 18) [22–39], Iraq (n = 2) [40, 41], Jordan (n = 4) [42–45], Kuwait (n = 3) [46–48], Lebanon (n = 1) [49], Libya (n = 2) [50, 51], Palestine (n = 2) [52, 53], Qatar (n = 2) [54, 55], Saudi Arabia (n = 14) [56–69], Syria (n = 4) [70–73], Turkey (n = 11) [74–84], UAE (n = 8) [85–92], and Yemen (n = 2) [93, 94]. No relevant published studies were found in Algeria, Bahrain, Morocco, Oman or Tunisia. The studies included a total of 94,491 participants between the ages of 12 months and 20 years. All the studies included both sexes, except four studies from Saudi Arabia where only males were included [59, 60, 62, 64]. The majority of the studies were cross-sectional studies (74 studies, 96.1%), two were longitudinal studies [76, 84] and one was a case–control study [40]. Approximately one-quarter of the studies (21/77) were published from 2000–2009, and the remaining 56 articles were published from 2010–2019. The majority of the included studies used the WHO indices (dmft, dmfs, DMFT, DMFS and their variations) as the scoring system. Other dental caries scoring systems, such as the American Association Paediatric Dentistry (AAPD), the Association of State and Territorial Dental Directors (ASTDD), the British Association for the Study of Community Dentistry (BASCD) and the International Caries Detection and Assessment System (ICADS), were also used for the assessment of ECC and dental caries.

Tables 2, 3, 4 and 5 show statistically significant determinants/risk factors contributing to dental caries derived from 76 studies. With regards to the influence of gender on caries prevalence, one article from Yemen, which assessed 90 children aged 5–15 years, found a dental caries prevalence of 40.7% and 75.0% in girls and boys, respectively [94]. Since no significant associations with BMI, the investigated determinant, were found, the study and its assessed variables were not presented in the tables [94]. Potential determinants that were investigated in the 76 studies that were found to be non-statistically significant by the authors of each of the articles were also not included in the tables. Moreover, for each study, the significant determinants/risk factors that had the highest level of statistical analysis are reported in the tables, i.e., if the author/s conducted either a univariate or bivariate analysis as the highest level of analysis, determinants that were found statistically significant for that analysis are reported in the tables. Finally, if the authors conducted a multivariate analysis as the highest level of analysis, only determinants that were found statistically significant in these analyses are reported in the tables, i.e., if determinants were statistically significant in uni- or bivariate analyses did not remain significant in a multivariate analysis, they are not included in the tables.

**Table 2:** Statistically significant determinants related to children’s sex, age and weight status contributing to dental caries

Determinants	Association: positive ( +), negative (−)^a^	Author study design	Country	Type of dentition	N	Age group (gender)	Study setting	Scoring system	Type/s of statistical analysis	Dental caries/scoring results
*Gender*										
Male (primary dentition)	+	Abbass et al. [20] (CS)	Egypt	Primary Mixed Permanent	369	3–18 y (M, F)	Clinic	WHO (dmft, deft, DMFT)	Kruskal–Wallis, Spearman’s	DCP = 74% dmft = 3.23 (SD 4.07) deft = 4.21 (SD 3.21) DMFT = 1.04 (SD 1.56)
Male	+	Kabil & Eltawil, 2016 [18] (CS)	Egypt	Primary	140	2–4 y (M, F)	Clinic	WHO AAPD-ECC	Logistic regression	DMFT = 9.96
Male	+	Kabil & Eltawil [19] (CS)	Egypt	Primary	108	2–4 y (M, F)	Clinic	WHO	Logistic regression	ECCP = 57% (2–3 y) ECCP = 73% (3–4 y)
Male	+	Abu Hamila [21](CS)	Egypt	Primary	560	1–3.5 y (M, F)	Clinic	WHO (dmft)	Chi-Square	ECCP = 69.6% dmft = 2.1–7.6
Male	+	Bayat-Movahed et al. [27] (CS)	Iran	Primary Permanent	18,946	3,6,9,12 y (M, F)	Community health centres	WHO (dmft, DMFT)	T-test Z-test	dmft = 1.9 (3 y) dmft = 5.0 (6 y) dmft = 3.6 (9 y) dmft = 0.6 (12 y) DMFT = 0.2 (6 y) DMFT = 0.9 (9 y) DMFT = 1.9 (12 y)
Male	+	Sadeghi et al. [35] (CS)	Iran	Permanent	747	12–15 y (M, F)	School	WHO (DMFT)	T-test, Chi-Square	Caries free = 16.1% DMFT = 2.83 (SD 2.2)
Male	+	Saied-Moallemi et al. [36] (CS)	Iran	Primary Permanent	459	9 y (M, F)	School	WHO (dmft, DMFT)	One-way ANOVA, Kruskal–Wallis, Mann- Whitney	dmft = 4.2 (M) dmft = 3.4 (F) DMFT = 0.4
Male	+	Goodson et al. [47] (CS)	Kuwait	Primary Mixed Permanent	8,319	Mean age = 11.36 y (grade 4 and 5) (M, F)	School	Percentage of decayed or filled teeth^b^	Multivariate rank-based Wilcoxon regression	*Decayed or filled teeth *(*all body weights*) = 11.01% (SEM 0.11) *Decayed or filled teeth *(*males*) = 11.76% (SEM 0.19) *Decayed or filled teeth* (*females*) = 10.53% (SEM 0.14)
Male	+	Hashim et al. [85] (CS)	UAE	Primary	1036	5,6 y (M, F)	School	WHO (dmft, dmfs)	Chi-Square, ZINB regression	DCP = 76.1% dmft = 4.4 dmfs = 10.2
Female	+	Bashirian et al. [26] (CS)	Iran	Primary Permanent	988	7–12 y (M, F)	School	WHO (dmft, DMFT)	Multiple regression	DCP = 80.36% dmft = 3.61 DMFT = 0.79
Female	+	Khani-Varzegani et al. [31] (CS)	Iran	Primary	756	4–7 y (M, F)	School	WHO (dmft)	Multivariate analysis	dmft median (25th–75th percentile): All = 4(2–8) Males = 4(2–9) Females = 5(2–8)
Female	+	Jahani et al. [30] (CS)	Iran	Primary Permanent	845	9 y (M, F)	School	WHO (dmft, DMFT)	Ordinal logistic regression	Moderate to high DCP^c^ = 50% of the children
Female	+	Farsi & Elkhodary [65] (CS)	KSA	Permanent	801	Mean age = 16.5 y (Grade 11) (M, F)	School	ASTDD (DT)	Mann- Whitney	DT boys = 3.9 (SD 3.5) DT girls = 4.9 (SD 3.7)
Female	+	Huew et al. [50] (CS)	Libya	Permanent	791	12 y (M, F)	School	WHO (DMFT, DMFS)	Multivariate analysis	DCP = 57.8% DMFT = 1.78 DMFS = 2.39
Female	+	Bener et al. [55] (CS)	Qatar	Permanent	1284	6–15 y (M, F)	Clinic	WHO (DMFT)	Multivariate analysis	DCP = 73% DMFT = 4.5
Gender	Unclear	Khadri et al. [90] (CS)	UAE	Permanent	803	11–17 y (M, F)	School	WHO (DMFT)	Multivariate regression	DCP = 75% DMFT = 3.19 (SD 2.9)
*Age*										
Age	+	Abbass et al. [20] (CS)	Egypt	Primary Mixed Permanent	369	3–18 y (M, F)	Clinic	WHO (dmft, deft, DMFT)	Kruskal–Wallis, Spearman’s	DCP = 74% dmft = 3.23 (SD 4.07) deft = 4.21 (SD 3.21) DMFT = 1.04 (SD 1.56)
Age	+	Abu Hamila [21] (CS)	Egypt	Primary	560	1–3.5 y (M, F)	Clinic	WHO (dmft)	Chi-Square	ECCP = 69.6% dmft = 2.1–7.6
Age	+	Bashirian et al. 2018 [26] (CS)	Iran	Primary Permanent	988	7–12 y (M, F)	School	WHO (dmft, DMFT)	Multiple regression	DCP = 80.36% dmft = 3.61 DMFT = 0.79
Age	+	Shaghaghian et al. [37] (CS)	Iran	Primary	396	3–6 y (M, F)	School	WHO (dmft)	Multivariate analysis	DCP = 69.9% dmft = 3.88
Age	+	Khani-Varzegani et al. [31] (CS)	Iran	Primary	756	4–7 y (M, F)	School	WHO (dmft)	Multivariate analysis	Median (25th–75th percentile) dmft: All = 4 (2–8) Boys = 4 (2–9) Girls = 5 (2–8)
Age	+	Eslamipour et al. [28] (CS)	Iran	Permanent	748	11–20 y (M, F)	School	WHO (DMFT)	Chi-Square, Binary logistic regression	DCP = 88.8% DMFT (11–14 y) = 4.94 (SD 3.59) DMFT (11–14 y) = 3.02 (SD 2.51) DMFT = 5.00 (SD 3.37) (14–17 y) DMFT (17–20 y) = 6.66 (SD 3.82)
Age	+	Mohebbi et al. [33] (CS)	Iran	Primary	504	12–36 m (M, F)	Clinic	WHO (dmft)	Logistic regression	ECCP: 12–15 m = 3% 16–19 m = 9% 20–25 m = 14% 26–36 m = 33% dmft = < 0.1 (12–15 m) dmft = 0.2 (16–19 m) dmft = 0.4(20–25 m) dmft = 1.2(26–36 m)
Age	+	Askarizadeh & Siyonat [23] (CS)	Iran	Primary	620	2–6 y (M, F)	School	WHO (dmft)	Chi-Square	DCP = 17.2% dmft = 8.5 (M) dmft = 7.8 (F)
Age	+	Sayegh et al. 2002^d^ [43] (CS) Sayegh et al. ^d^ [45] (CS)	Jordan	Primary	1140	4–5 y (M, F)	School	WHO (dmft)	Multivariate analysis	DCP = 67% dmft > 4 in 31%
Age	+	Al-Malik et al. [57] (CS)	KSA	Primary	987	2–5 y (M, F)	School	BASCD	Stepwise multiple logistic regression	DCP = 73% ECCP = 43% dmft = 4.8 dmfs = 12.7
Age	+	Wyne et al. [69] (CS)	KSA	Primary	1016	2–6 y (M, F)	School	WHO (dmft)	Logistic regression	DCP = 27.3% dmft = 8.6
Age	+	Al-Mutawa el al. [46] (CS)	Kuwait	Primary Permanent	4588	5,6,12,14 y (M, F)	School	WHO (dft, DMFT, DFS)	Multivariate analysis	dft = 4.6 (5–6 y) DMFT = 0.4(6 y) DMFT = 2.6 (12 y) DMFT = 3.9 (14 y) DFS = 0.4 (6 y) DFS = 3.4 (12 y) DFS = 5.2 (14 y)
Age	+	Qadri et al. [73] (CS)	Syria	Primary	400	3–5 y (M, F)	School	ECC WHO (dmft, dmfs)	Logistic regression	ECCP = 48% DCP = 70% dmft = 4.25 (SD 4.24)
Age	+	İnan-Eroğlu et al. [78] (CS)	Turkey	Primary	395	36–71 m (M, F)	School	WHO (dmft, dmfs)	Mann–Whitney, Kruskal–Wallis	dmft = 4.7 dmfs = 8.0
Age	+	Dogan et al. [77] (CS)	Turkey	Primary	3171	8–60 m (M, F)	Clinic	WHO (dft)	Chi-Square	ECCP = 17.3% dft = 0.63 (1.79)
Age	+	Namal et al. [80] (CS)	Turkey	Primary	598	3–6 y (M, F)	School	WHO (dft)	Multiple logistic regression	dft = 74.1%
Age	+	Olmez et al. [82] (CS)	Turkey	Primary	95	9–57 m (M, F)	Clinic	WHO (dft)	Chi-Square, Kruskal–Wallis	DCP = 75.5% dft = 6.2
Age	+	Bener et al. [55] (CS)	Qatar	Permanent	1284	6–15 y (M, F)	Clinic	WHO (DMFT)	Multivariate analysis	DCP = 73% DMFT = 4.5
Age	Unclear	Khadri et al. [90] (CS)	UAE	Permanent	803	11–17 y (M, F)	School	WHO (DMFT)	Multivariate regression	DCP = 75% DMFT = 3.19 (SD 2.9)
Age	+	Hashim et al. [85] (CS)	UAE	Primary	1036	5,6 y (M, F)	School	WHO (dmft, dmfs)	Chi-Square,ZINB regression	DCP = 76.1% dmft = 4.4 dmfs = 10.2
*Weight status*										
Over weight	+	Jahani et al. [30] (CS)	Iran	Primary Permanent	845	9 y (M, F)	School	WHO (dmft/DMFT)	Ordinal logistic regression	Moderate to high DCP^1^ = 50% of the children
BMI	+	Bagherian & Sadeghi [25] (CS)	Iran	Primary	400	30–70 m (M, F)	Not specified	WHO (defs)	Multiple logistic regression	ECCP = 55.2% S-ECCP = 51.2% defs = 8.37 (SD 11.2)
BMI	+	Abu El Qomsan et al. [56] (CS)	KSA	Permanent	386	6–12 y (M, F)	School and Clinic	WHO (DMFT, DT, FT)	One-way ANOVA, Spearman’s	*DT:* Underweight = 3.06 (SD 1.48) Normal weight = 2.90 (SD 2.34) Over weight = 3.69 (SD 2.39) Obese = 4.00 (SD 2.57) *FT:* Underweight = 0.25 (SD 0.68) Normal weight = 0.34 (SD 0.95) Over weight = 0.39 (SD 0.70) Obese = 0.68 (SD 1.18)
BMI	−	Alghamdi & Almahdy [59] (CS)	KSA	Permanent	610	14–16 y (M)	School	Not specified DMFT	Logistic regression	DCP = 54.1%
Low BMI	+	Quadri et al. [68] (CS)	KSA	Primary Permanent	360	6–15 y (M, F)	School	WHO (dft/DMFT)	Logistic regression	dft/DMFT = 2.52 (F), 1.88 (M)
BMI	−	Goodson et al. [47] (CS)	Kuwait	Primary Mixed Permanent	8,319	Mean age = 11.36 y (grade 4 & 5) (M, F)	School	Percentage of decayed or filled teeth^1^	Multivariate rank-based Wilcoxon regression	*Decayed or filled* teeth (all body weights) = 11.01% (SEM 0.11) *Decayed or filled teeth *(*males*) = 11.76% (SEM 0.19) *Decayed or filled teeth *(*females*) = 10.53% (SEM 0.14)
Under weight	+	Köksal et al. [79] (CS)	Turkey	Primary Permanent	245	5–6 y (M. F)	Unclear	WHO (dmft, DMFT, dmfs)	Chi-Square, Mann- Whitney, Spearman’s	DCP = 85.9% dmft = 5.3 (SD 3.78) DMFT = 0.27(SD 0.74) dmfs = 10.5(SD 9.67) DMFS = 0.33(SD 0.95)
Weight status	Varied^e^	Bhayat et al. [64] (CS)	KSA	Permanent	402	12 y (M)	School	WHO (DMFT)	Linear regression	DCP = 49% DMFT = 1.46 (SD 2.04)
BMI	+	Bener et al. [55] (CS)	Qatar	Permanent	1284	6–15 y (M, F)	Clinic	WHO (DMFT)	Multivariate analysis	DCP = 73% DMFT = 4.5

*AAPD* American Association Paediatric Dentistry, *BASCD* British Association for the Study of Community Dentistry, *CS* Cross-sectional, *CC* Case control, *DCP* Dental caries prevalence, *deft* decayed, extracted due to caries and filled primary teeth, *dfs* decayed, filled surfaces in primary teeth, *dft* decayed, filled primary teeth, *dmfs* decayed, missing and filled surfaces in primary teeth; *DMFS* decayed, missing and filled surfaces in permanent teeth, *dmft* decayed, missing, filled primary teeth, *DMFT* decayed, missing, filled permanent teeth, *ECC* Early childhood caries, *ECCP* Early childhood caries prevalence, *F* Female, *ICADS* The international caries Detection and Assessment System, *L* Longitudinal, *KSA* Kingdom of Saudi Arabia, *m* months, *M* Male, *WHO* World Health Organisation, *SiC* Significant caries index, *SD* standard deviation, *y* years

^a^Association: Positive ( +), negative (−) refers to this factor being either a statistically significant risk factor for caries (positive, +) or to this factor being statistically significant protective against caries (negative, −). In some studies it could not be determined whether a factor was positively or negatively associated with caries and in these cases the relation is described as unclear

^b^The author calculated this as follows the decayed or filled teeth (%) = 100 × [(number of primary teeth with fillings) + (number of permanent teeth with fillings) + (number of decayed primary teeth) + (number decayed permanent teeth)]/[(number of primary teeth) + (number of permanent teeth)]

^c^The children were categorized into three groups on the basis of WHO caries severity classification. Low caries level was defined as dmft/DMFT ≤ 2.6, moderate caries as dmft/DMFT of 2.7–4.4 and high caries as dmft/DMFT > 4.4

^d^Sayegh et al. 2002 and Sayegh et al. 2005 seem to be based on the same study population and the results mentioned in this table, have been reported in both articles

^e^Normal weight status-positive association to caries, whereas the caries prevalence was lower in under and overweight children

**Table 3:** Statistically significant socio-economic, socio-demographic, school type and geographical-related determinants contributing to dental caries

Determinants	Association: positive ( +), negative (−)^a^	Author study design	Country	Type of dentition	N	Age group (gender)	Study setting	Scoring system	Type/s of statistical analysis	Dental caries/scoring results
*Mother’s attributes*										
Mother’s education	–	Abu Hamila [21] (CS)	Egypt	Primary	560	1–3.5 y (M, F)	Clinic	WHO (dmft)	Chi-Square	ECCP = 69.6% dmft = 2.1–7.6
Mother’s education	–	Bashirian et al. [26] (CS)	Iran	Primary Permanent	988	7–12 y (M, F)	School	WHO (dmft, DMFT)	ANOVA	DCP = 80.36% dmft = 3.61 DMFT = 0.79
Mother’s education	–	Shaghaghian et al. [37] (CS)	Iran	Primary	396	3–6 y (M, F)	School	WHO (dmft)	Multivariate analysis	DCP = 69.9% dmft = 3.88
Mother’s education	–	Haghdoost et al. [29] (CS)	Iran	Primary Permanent	8725	6 y (M, F)	Clinic	WHO	Linear regression, Logistic regression	DCP = 87%
Mother’s education	–	Khani-Varzegani et al. [31](CS)	Iran	Primary	756	4–7 y (M, F)	School	WHO (dmft)	Multivariate analysis	dmft median (25th–75th percentile): All = 4(2–8) Males = 4(2–9) Females = 5(2–8)
Mother’s education (low levels)	+	Alhabdan et al. [60] (CS)	KSA	Primary	578	6–8 y (M, F)	School	WHO (dmft)	Adjusted odds ratios Multivariate model logistic regression	DCCP = 83% dmft 4.20 (SD 2.96)
Mother’s education	–	Al-Meedani [58] (CS)	KSA	Primary	388	3–5 y (M, F)	School	WHO (dmft, dmfs)	Chi-Square Z-test	DCP = 69% dmft = 3.4 dmfs = 6.9
Mother’s education	–	Quadri et al. [67] (CS)	KSA	Primary Permanent	853	6–15 y (M, F)	School	WHO (dft, DMFT)	Multi regression	DCP = 91.3%
Mother’s education	–	Al-Malik et al. [57] (CS)	KSA	Primary	987	2–5 y (M, F)	School	BASCD (dmft, dmfs)	Stepwise multiple logistic regression	DCP = 73% Rampant caries = 43% dmft = 4.8 dmfs = 12.7
Mother’s education (number of filled teeth in the child)	+	Azizi et al. [52] (CS)	Palestine	Primary	1376	4–6 y (M, F)	Clinic	WHO (dmft)	Not indicated	DCP = 76% dmft = 2.46
Mother’s education	–	Ozer et al. [83] (CS)	Turkey	Primary	226	3–6 y (M, F)	School	WHO (dmft) AAPD	Bivariate analysis	ECCP = 46.9% dmft = 2.87
Mother’s education	–	Namal et al. [81] (CS)	Turkey	Primary	542	5–6 y (M, F)	School	WHO (dmft)	Multiple logistic regression	DCP = 76.8% dmft = 3.74 (3.49) SiC = 7.75 (2.56)
Mother’s education	–	Elamin et al. [89] (CS)	UAE	Primary	186	1.5–4 y (M, F)	School	WHO (dmft)	T-test, Pearson-s	DCP: 41% dmft:1.7 ± 2.81
Mother’s occupation (Employed)	+	Abu Hamila [21] (CS)	Egypt	Primary	560	1–3.5 y (M, F)	Clinic	WHO (dmft)	Chi-Square	ECCP = 69.6% dmft = 2.1–7.6
Mother’s occupation (not employed)	+	Amin & Al-Abad [62] (CS)	KSA	Permanent	1115	10–14 y (M)	School	WHO	Stepwise logistic regression	DCP = 68.9%
Mother’s caries experience	+	Kabil & Eltawil [18] (CS)	Egypt	Primary	140	2–4 y (M, F)	Clinic	WHO (DMFT) AAPD	Logistic regression	DMFT = 9.96
Mother’s current caries experience	+	Kabil & Eltawil [19] (CS)	Egypt	Primary	108	2–4 y (M, F)	Clinic	WHO	Logistic regression	ECCP = 57% (2–3 y), 73% (3–4 y)
*Father’s attributes*										
Father’s education (CAST score of ≥ 3 in primary molar teeth)	−	Babaei et al. [24] (CS)	Iran	Primary & Permanent molar teeth	739	6–7 y (M, F)	School	CAST index^b^	Multivariate logistic regression	*Permanent molars:* Healthy status in 89.3–93.7% of the teeth *Primary molars:* Morbidity status in 25.3 to 31.2% of the teeth Serious morbidity status with Pulp involvement in 2.9–10.5% of the teeth and abscess/fistula in < 1% of the teeth
Father’s education	−	Bayat-Movahed et al. [27] (CS)	Iran	Primary Permanent	18,946	3,6,9,12 y (M, F)	Community health centres	WHO (dmft, DMFT)	T-test, Z test	dmft = 1.9 (3 y) dmft = 5.0 (6 y) dmft = 3.6 (9 y) dmft = 0.6 (12 y) DMFT = 0.2 (6 y) DMFT = 0.9 (9 y) DMFT = 1.9 (12 y)
Father’s Education	−	Huew et al. [50] (CS)	Libya	Permanent	791	12 y (M, F)	School	WHO (DMFT, DMFS)	Multivariate analysis	DCP = 57.8% DMFT = 1.78 DMFS = 2.39
Father’s Education	Unclear	Khadri et al. [90] (CS)	UAE	Permanent	803	11–17 y (M, F)	School	WHO (DMFT)	Multivariate regression	DCP = 75% DMFT = 3.19 (SD 2.9)
Father’s Occupation	+	Shaghaghian et al. [37] (CS)	Iran	Primary	396	3–6 y (M, F)	School	WHO (dmft)	Multivariate analysis	DCP = 69.9% dmft = 3.88
Father’s occupation (Low educational occupations)	+	Namal et al. [80] (CS)	Turkey	Primary	598	3–6 y (M, F)	School	WHO (dft)	Multiple logistic regression	DCP = 74.1%
Father’s occupation (Self-employment)	+	Amanlou et al. [22] (CS)	Iran	Primary Permanent	205	3–6 y (M, F)	School	WHO (DMFT)^c^	Stepwise multiple regression	DCP = 49.3% DMFT = 0.99 (SD 0.13)
*Parents attributes*										
Parents’ education (primary dentition)	−	Abbass et al. [20] (CS)	Egypt	Primary Mixed Permanent	369	3–18 y (M, F)	Clinic	WHO (dmft, deft, DMFT)	Kruskal–Wallis, Spearman’s	DCP = 74% dmft = 3.23 (SD 4.07) deft = 4.21 (SD 3.21) DMFT = 1.04 (SD 1.56)
Parents’ education level	-	Sistani et al. [38] (CS)^d^	Iran	Primary	2080	3–6 y (M, F)	School	WHO (dmft)	T-test, ANOVA	ECCP varied between 51.1 and 71.9% during 2007–2015 dmft = 4.01 (SD 3.89)
Socio-economic factors^e^	+	Ahmed et al. [41] (CS)	Iraq	Permanent	392	12 y (M, F)	School	WHO (DMFT)	ANOVA	DCP = 62% DMFT = 1.7
Parents’ Education	−	Al-Mendalawi & Karam, 2014 [40] (CC)	Iraq	Primary	684	< 6 y (M, F)	Clinic	WHO (DMFT)^f^	Chi-Square	DMFT = 2.03
Parents Education	−	Rajab et al. [42] (CS)	Jordan	Primary Permanent	2496 (6 y) 2560 (12 y)	6 y, 12 y (M, F)	School	WHO (dmft, DMFT)	Multivariate analysis linear regression	DCP = 76.4% (6 y) DCP = 45.5% (12 y) dmft = 3.3 (6 y) DMFT = 1.1 (12 y)
Parents’ employment status	−	Sistani et al. [38] (CS)^d^	Iran	Primary	2080	3–6 y (M, F)	School	WHO (dmft)	T-test, ANOVA	ECCP varied between 51.1 and 71.9% during 2007–2015 dmft = 4.01 (SD 3.89)
Parents’ employment status	−	Khodadadi et al. [32] (CS)	Iran	Primary	384	21–84 m (M, F)	Not specified	WHO (dmft)	Multiple linear regression	dmft = 8.2
Socio-economic status^g^	−	Abbass et al. [20] (CS)	Egypt	Primary Mixed Permanent	369	3–18 y (M, F)	Clinic	WHO (dmft, deft, DMFT)	Kruskal–Wallis, Spearman’s	DCP = 74% dmft = 3.23 (SD 4.07) deft = 4.21 (SD 3.21) DMFT = 1.04 (SD 1.56)
Family affluent scale	−	Khani-Varzegani et al. [31] (CS)	Iran	Primary	756	4–7 y (M, F)	School	WHO	Multivariate analysis	dmft median (25th–75th percentile): All = 4(2–8) Boys = 4(2–9) Girls = 5(2–8)
Income	−	Al-Mendalawi & Karam [40] (CC)	Iraq	Primary	684	< 6 y (M, F)	Clinic	WHO (DMFT)^f^	Chi-Square	DMFT = 2.03
Low family income	+	Alhabdan et al. [60] (CS)	KSA	Primary	578	6–8 y (M)	School	WHO (dmft)	Adjusted Odds Ratios, Multivariate model logistic regression	DCCP = 83% dmft 4.20 (SD 2.96)
Lack of dental insurance-	+	Alhabdan et al. [60] (CS)	KSA	Primary	578	6–8 y (M)	School	WHO (dmft)	Adjusted Odds Ratios, Multivariate model logistic regression	DCCP = 83% dmft 4.20 (SD ± 2.96)
Socio-Economic Status^h^	−	Alghamdi & Almahdy [59] (CS)	KSA	Permanent	610	14–16 y (M)	School	Not specified (DMFT)	Logistic regression	DCP = 54.1%
Socio-Economic Status^i^	−	Rajab et al. [42] (CS)	Jordan	Primary Permanent	2496 (6 y) 2560 (12 y)	6 y, 12 y (M, F)	School	WHO (dmft, DMFT)	Multivariate analysis linear regression	DCP = 76.4% (6 y) DCP = 45.5% (12 y) dmft = 3.3 (6 y) DMFT = 1.1 (12 y)
Household income	+	Bener et al. [55] (CS)	Qatar	Permanent	1284	6–15 y (M, F)	Clinic	WHO (DMFT)	Multivariate analysis	DCP = 73% DMFT = 4.5
House Hold Income	−	Hashim et al. [86] (CS)	UAE	Primary	1036	3–6 y (M, F)	School	WHO	Logistic regression	Severe ECCP = 31.1%
*Family demographic*										
Sibling order	Varied^j^	Abu Hamila [21] (CS)	Egypt	Primary	560	1–3.5 y (M, F)	Clinic	WHO (dmft)	Chi-Square	ECCP = 69.6% dmft = 2.1–7.6
Number of Siblings	+	Shaghaghian et al. [37] (CS)	Iran	Primary	396	3–6 y (M, F)	School	WHO (dmft)	Multivariate analysis	DCP = 69.9% dmft = 3.88
Large family size	+	Al-Meedani [58] (CS)	Iraq	Primary	684	0–6 y (M, F)	Clinic	WHO (dmft, dmfs)	Chi-Square, Z-test	DCP = 69% dmft = 3.4 dmfs = 6.9
Large family size	+	Amin & Al-Abed [62] (CS)	KSA	Permanent	1115	10–14 y (M)	School	WHO	Stepwise logistic regression	DCP = 68.9%
Nationality (Emirati)	+	Elamin et al. [89] (CS)	UAE	Primary	186	1.5–4 y (M, F)	School	WHO (dmft)	T-tests Pearson’s	DCP = 41% dmft = 1.7 (SD 2.81)
Geographical Location	Varied^k^	Al Mutawa et al. [48] (CS)	Kuwait	Primary	1277	4 &5 y (M, F)	School	WHO	T-test Chi Square	dft/dfs = 3.7/6.9 (4 y) dft/dfs = 4.8/9.6 (5 y)
Geographical Location	Varied^l^	Ballouk & Dashash 2019 [70] (CS)	Syria	Primary Permanent	1500	8–12 y (M, F)	School	WHO (DMFT, dmft)	ANOVA Chi-Square	DCP = 79.1% dmft = 2.47 (SD 2.94) DMFT = 2.03 (SD 1.81)
Rural living	+	Al-Mendalawi & Karam [40] (CC)	Iraq	Primary	684	< 6 y (M, F)	Clinic	WHO (DMFT)^f^	Chi-Square	DMFT = 2.03
Rural living	+	Elamin et al. [89] (CS)	UAE	Primary	186	1.5–4 y (M, F)	School	WHO (dmft)	T-test, -Pearson’s	DCP = 41% Dmft = 1.7 (SD 2.81)
Urban living	+	Bayat-Movahed et al. [27] (CS)	Iran	Primary Permanent	18,946	3,6,9,12 y (M, F)	Community health centres	WHO	T-test Z-test	dmft = 1.9 (3 y) dmft = 5.0 (6 y) dmft = 3.6 (9 y) dmft = 0.6 (12 y) DMFT = 0.2 (6 y) DMFT = 0.9 (9 y) DMFT = 1.9 (12 y)
Semi-urban living	+	Al- Darwish et al. [54] (CS)	Qatar	Permanent	2113	12–14 y (M, F)	School	WHO (DMFT)	Multinomial logistic regression, Adjusted Odds Ratio	DCP = 85% DMFT (12 y) = 4.62 (SD 3.2) DMFT (13 y) = 4.79 (SD 3.5) DMFT (14 y) = 5.51 (SD 3.7)
*School type*										
Public Schools	+	Farsi & Elkhodary [65] (CS)	KSA	Permanent	801	Mean age = 16.5 y (Grade 11) (M, F)	School	ASTDD (DT)	Mann- Whitney	DT boys = 3.9 (SD 3.5) DT girls = 4.9 (SD 3.7)
Public Schools	+	Al-Malik et al. [57] (CS)	KSA	Primary	987	2–5 y (M, F)	School	BASCD (dmft, dmfs)	Stepwise multiple logistic regression	DCP = 73% Rampant caries = 43% dmft = 4.8 dmfs = 12.7
Private schools	−	Sgan-Cohen et al. [53] (CS)	Palestine	Permanent	286	12 y (M, F)	School	WHO (DMFT)	Multivariate analysis	DMFT = 1.98
Public schools	+	Cinar & Murtomaa [74] (CS)	Turkey	Permanent	611	10–12 y (M, F)	School	WHO (DMFS)	T-test Chi-Square Logistic regression	DMFS = 4.44 (public school) DMFS = 2.64 (private school)
Public schools	+	Cinar & Murtromaa [75] (CS)	Turkey^m^	Permanent	611	10–12 y (M, F)	School	WHO (DMFT)	T-test Logistic regression	DMFT = 2.93

*AAPD* American Association Paediatric Dentistry, *BASCD* British Association for the Study of Community Dentistry, *CS* Cross-sectional, *CC* Case control, *DCP* Dental caries prevalence, *deft* decayed, extracted due to caries and filled primary teeth, *dfs* decayed, filled surfaces in primary teeth, *dft* decayed, filled primary teeth, *dmfs* decayed, missing and filled surfaces in primary teeth; *DMFS* decayed, missing and filled surfaces in permanent teeth, *dmft* decayed, missing, filled primary teeth, *DMFT* decayed, missing, filled permanent teeth, *ECC* Early childhood caries, *ECCP* Early childhood caries prevalence, *F* Female, *ICADS* The international caries Detection and Assessment System, *L* Longitudinal, *KSA* Kingdom of Saudi Arabia, *m* months *M* Male, *WHO* World Health Organisation, *SiC* Significant caries index, *SD* Standard deviation, *y* years

^a^Association: Positive ( +), negative (−) refers to this factor being either a statistically significant risk factor for caries (positive, +) or to this factor being statistically significant protective against caries (negative, −). In some studies it could not be determined whether a factor was positively or negatively associated with caries and in these cases the relation is described as unclear

^b^The CAST index scoring system is as follows: “0: sound”, “1: sealant”, “2: restoration”, “3: enamel lesions”, “4, 5: dentine lesions”, “6: pulp involvement”, “7: abscess/fistula”, “8: tooth loss”. If a situation did not match any codes from 0 to 8, a code 9 was assigned. The codes 0–2, 3, 4–5, 6–7, and 8 were considered as “healthy”, “pre-morbidity”, “morbidity”, “serious morbidity”, and “mortality”, respectively

^c^The authors describe their scoring as WHO (DMFT) whereas it should be noted that the age group is 3–6 year olds where normally WHO (dmft) is being used

^d^Data was collected during 9 years. In each year data was collected in a new sample

^e^The mean FT score was significantly higher for children having mothers with higher education, fathers with higher education and for residents of higher socio-economic areas, as compared to their counterparts in the opposite groups

^f^The authors describe their scoring as WHO(DMFT) whereas it should be noted that the age group is 0–6 year olds where normally WHO (dmft) is being used

^g^The SES level was based on the level of parental education and its type, guardians’ occupation and address

^h^SES score based on parental education and suburban location of residence

^i^SES score based on school type: low SES: deprived areas and refugee camps, medium SES: state schools, high SES: private schools

^j^The sibling order impacts dental caries status: 84.44%, 74,37%, 40.19% and 77.65% of only, eldest, middle and youngest child/ren had dental caries, respectively

^k^Dental caries prevalence differed between the 6 different regions/governorates in Kuwait but the characteristics of the regions are not described

^l^Dental caries prevalence differed between different parts/regions in Damascus but the characteristics of the regions are not described

^m^A comparative study with Finland

**Table 4:** Statistically significant dental related determinants/risk factors contributing to dental caries

Determinants	Association: positive ( +), negative (−)^a^	Author, year (study design)	Country	Type of dentition	N	Age group (gender)*	Study setting	Scoring system	Type/s of statistical analysis	Dental caries/scoring system
*Tooth brushing frequency*										
Tooth brushing-frequent (Primary, mixed)	−	Abbas et al. [20] (CS)	Egypt	Primary Mixed Permanent	369	3–18 y (M, F)	Clinic	WHO (dmft, deft, DMFT)	Kruskal–Wallis, Spearman’s	DCP = 74% dmft = 3.23 (SD 4.07) deft = 4.21 (SD 3.21) DMFT = 1.04 (SD 1.56)
Tooth brushing-frequent	−	Amanlou et al. [22] (CS)	Iran	Primary Permanent	205	3–6 y (M, F)	School	WHO (DMFT)^b^	Stepwise multiple regression	DCP = 49.3% DMFT = 0.99 (SD 0.13)
Tooth brushing-frequent	−	Shaghaghian et al. [37] (CS)	Iran	Primary	396	3–6 y (M, F)	School	WHO (dmft)	Multivariate analysis	DCP = 69.9% dmft = 3.88
Tooth brushing-frequent	−	Al-Mendalawi & Karam [40] (CC)	Iraq	Primary	684	< 6 y (M, F)	Clinic	WHO (dmft)	Chi-Square	dmft = 2.03
Tooth brushing-frequent	−	Bener et al. [55] (CS)	Qatar	Permanent	1284	6–15 y (M, F)	Clinic	WHO (DMFT)	Multivariate analysis	DCP = 73% DMFT = 4.5
Tooth brushing-frequent	−	Namal et al. [81] (CS)	Turkey	Primary	542	5–6 y (M, F)	School	WHO (dmft)	Multiple logistic regression	DCP = 76.8% dmft = 3.74 (SD 3.49) SiC = 7.75 (SD 2.56)
Tooth brushing-frequent	−	Tulunoğlu et al. [84] (L)^c^	Turkey	Primary Permanent	733	6–8 y (M, F)	School	WHO (dfs, DFS)	Chi-Square	dfs Baseline: GI:2.79, GII:3.12, GIII: 2.9 Dfs Final: GI: 2.14, GII:3.79, GIII: 3.69 DFS Baseline: GI: 0.16, GII: 0.20, GIII: 0.15 DFS Final: GI: 0.79, GII: 0.80 GIII: 1.46
Tooth brushing-frequent	−	Elamin et al. [89] (CS)	UAE	Primary	186	1.5–4 y (M, F)	School	WHO (dmft)	T-test, Pearson’s	DCP: 41% dmft:1.7 (SD 2.81)
Tooth brushing-frequent	−	Kowash et al. [91] (CS)	UAE	Primary	540	4–6 y (M, F)	School	WHO (dmft)	Chi-Square	ECCP = 74.1% dmft = 3.01 SiC = 13.3
Tooth brushing -irregular or no brushing	+	Alhabdan et al. [60] (CS)	KSA	Primary	578	6–8 y (M)	School	WHO (dmft)	Adjusted Odds Ratios, Multivariate model logistic regression	DCP: 83% dmft = 4.20 (SD 2.96)
Tooth brushing -Irregular or no brushing	+	Paul [66] (CS)	KSA	Primary	103	5 y (M, F)	Clinic	WHO (dmft)	Chi-Square	DCP = 83.5% dmft = 7.1 (SD 5.7)
*Tooth brushing initiation age*										
Tooth brushing initiation -late	+	Alhabdan et al. [60] (CS)	KSA	Primary	578	6–8 y (M)	School	WHO (dmft)	Adjusted Odds Ratios, Multivariate model logistic regression	DCP: 83% dmft 4.20 (SD 2.96)
Tooth brushing initiation -late	+	Al-Malik et al. [57] (CS)	KSA	Primary	987	2–5 y (M, F)	School	BASCD	Stepwise multiple ogistic regression	DCP = 73% ECCP = 43% dmft = 4.8 dmfs = 12.7
*Tooth brushing with adult help and aid*										
Tooth brushing with adult help	−	Bashirian et al. [26] (CS)	Iran	Primary	988	7–12 y (M, F)	School	WHO (dmft, DMFT)	ANOVA	DCP = 80.36% dmft = 3.61 DMFT = 0.79
Tooth brushing with adult help	−	Al-Malik et al. [57] (CS)	KSA	Primary	987	2–5 y (M, F)	School	BASCD	Stepwise multiple logistic regression	DCP = 73% ECCP = 43% dmft = 4.8 dmfs = 12.7
Tooth brushing- with use of fluoridated toothpaste	−	Alghamdi & Almahdy [59] (CS)	KSA	Permanent	610	14–16 y (M)	School	Not specified	Logistic regression	DCP = 54.1%
*Oral hygiene and practices attributes*										
Oral hygiene^d^ (CAST score of ≥ 3 in primary molar teeth)	+	Babaei et al. [24] (CS)	Iran	Primary and Permanent molar teeth	739	6–7 y (M, F)	School	CAST index^e^	Multivariate logistic regression	*Permanent molars:* Healthy status in 89.3–93.7% of the teeth *Primary molars:* Morbidity status in 25.3 to 31.2% of the teeth Serious morbidity status with Pulp involvement in 2.9–10.5% of the teeth and abscess/fistula in < 1% of the teeth
Oral Hygiene-dental plaque presence	+	Mohebbi et al. [33] (CS)	Iran	Primary	504	12–36 m (M, F)	Clinic	WHO (dmft)	Logistic regression	ECCP: 12–15 m = 3% 16–19 m = 9% 20–25 m = 14% 26–36 m = 33% dmft = < 0.1 (12–15 m) dmft = 0.2 (16–19 m) dmft = 0.4(20–25 m) dmft = 1.2(26–36 m)
Oral hygiene-poor	+	Al-Mutawa el al. [46] (CS)	Kuwait	Primary Permanent	4588	5,6,12,14 y (M, F)	School	WHO (dft, DMFT, DFS)	Multivariate analysis	dft = 4.6 (5–6 y) DMFT = 0.4(6 y) DMFT = 2.6 (12 y) DMFT = 3.9 (14 y) DFS = 0.4 (6 y) DFS = 3.4 (12 y) DFS = 5.2 (14 y)
Oral hygiene-poor	+	Amin & Al-Abad [62] (CS)	KSA	Permanent	1115	10–14 y (M)	School	WHO	Stepwise logistic regression	DCP = 68.9%
Oral hygiene-poor	+	Dashash & Blinkhorn [71] (CS)	Syria	Primary	727	5 y (M, F)	School	WHO (dmft, DMFT)	Multiple logistic regression	DCP = 61% dmft = 3.27(3.71)
Oral hygiene-poor	+	Jaghasi et al. [72] (CS)	Syria	Not specified	504	6–12 y (M, F)	School	WHO	Logistic regression	DCP = 85%
Oral practices-poor	+	Kowash et al. [91] (CS)	UAE	Primary	540	4–6 y (M, F)	School	WHO (dmft)	Chi-Square	ECCP = 74.1% dmft = 3.01 SiC = 13.3
Not feeling embarrassed when smiling	−	Ahmed et al. [41] (CS)	Iraq	Permanent	392	12 y (M, F)	School	WHO (DMFT)	ANOVA	DCP = 62% DMFT = 1.7
Permanent dentition	+	Al-Mutawa el al. [46] (CS)	Kuwait	Primary Permanent	4588	5,6,12,14 y (M, F)	School	WHO (dft, DMFT, DFS)	Multivariate analysis	dft = 4.6 (5–6 y) DMFT = 0.4 (6 y) DMFT = 2.6 (12 y) DMFT-3.9 (14 y) DFS = 0.4 (6 y) DFS = 3.4 (12 y) DFS = 5.2 (14 y)
*Dental services visits attributes*										
Dental services-child’s first visit	−	Kabil & Eltawil [19] (CS)	Egypt	Primary	108	2–4 y (M, F)	Clinic	WHO	Logistic regression	ECCP = 57% (2–3 y), ECCP = 73% (3–4 y)
Dental visits-regular	−	Kabil and Eltawil [18] (CS)	Egypt	Primary	140	2–4 y (M, F)	Clinic	WHO AAPD-ECC	Logistic regression	DMFT = 9.96
Dental visits-regular	−	Alhumaid et al. [61] (CS)	KSA	Primary Permanent	921	6–12 y (M, F)	School	Basic screening survey	Multivariate analysis	DCP = 63.5%
Dental services -not attending for preventive measures	+	Dashash & Blinkhorn [71] (CS)	Syria	Primary	727	5 y (M, F)	School	WHO (dmft, DMFT)	Multiple logistic regression	DCP = 61% dmft = 3.27 (SD 3.71)
Dental visits- for pain complaints/dental problems	+	Shaghaghian et al. [37] (CS)	Iran	Primary	396	3–6 y (M, F)	School	WHO	Multivariate analysis	DCP = 69.9% dmft = 3.88
Dental visits- for pain complaints/dental problems	+	Alhabdan et al. [60] (CS)	KSA	Primary	578	6–8 y (M)	School	WHO (dmft)	Adjusted Odds Ratios, Multivariate model logistic regression	DCP: 83% dmft = 4.20 (SD 2.96)
Dental visits	Unclear	Khadri et al. [90] (CS)	UAE	Permanent	803	11–17 y (M, F)	School	WHO (DMFT)	Multivariate regression	DCP = 75% DMFT = 3.19 (SD 2.9)
*Parental oral health status and knowledge attributes*										
Parental dental caries status	+	Yazdani et al. 2018 [39] (CS)	Iran	Primary Permanent	258	5–15 y (M, F)	Clinic	WHO (dmft, DMFT)	Pearson’s	dmft = 6.33 (SD3.80) DMFT = 1.48 (SD1.90)
Parental knowledge on oral hygiene	−	Yazdani et al. [39] (CS)	Iran	Primary Permanent	258	5–15 y (M, F)	Clinic	WHO (dmft, DMFT)	Pearson’s	dmft = 6.33 (SD3.80) DMFT = 1.48 (SD1.90)
Mother’s caries experience	+	Kabil & Eltawil [18] (CS)	Egypt	Primary	140	2–4 y (M, F)	Clinic	WHO (DMFT) AAPD	Logistic regression	DMFT = 9.96
Mother’s current caries experience	+	Kabil & Eltawil [19] (CS)	Egypt	Primary	108	2–4 y (M, F)	Clinic	WHO	Logistic regression	ECCP = 57% (2–3 y), 73% (3–4 y)
Parental knowledge on oral hygiene	−	Kowash et al. [91] (CS)	UAE	Primary	540	4–6 y (M, F)	School	WHO (dmft)	Chi-Square	ECCP = 74.1% dmft = 3.01 SiC = 13.3

*AAPD* American Association Paediatric Dentistry, *BASCD* British Association for the Study of Community Dentistry, *CS* Cross-sectional, *CC* Case control, *DCP* Dental caries prevalence, *deft* decayed, extracted due to caries and filled primary teeth, *dfs* decayed, filled surfaces in primary teeth, *dft* decayed, filled primary teeth, *dmfs* decayed, missing and filled surfaces in primary teeth; *DMFS* decayed, missing and filled surfaces in permanent teeth;

*dmft* decayed, missing, filled primary teeth, *DMFT* decayed, missing, filled permanent teeth, *ECC* Early childhood caries, *ECCP* Early childhood caries prevalence, *F* Female, *ICADS* The international caries Detection and Assessment System, *L* Longitudinal, *KSA* Kingdom of Saudi Arabia, *m* months *M* Male, *WHO* World Health Organisation, *SiC* Significant caries index, *SD* Standard deviation, *y* years

^a^ Association: Positive ( +), negative (−) refers to this factor being either a statistically significant risk factor for caries (positive, +) or to this factor being statistically significant protective against caries (negative, −). In some studies it could not be determined whether a factor was positively or negatively associated with caries and in these cases the relation is described as unclear

^b^The authors describe their scoring as WHO(DMFT) whereas it should be noted that the age group is 3–6 year olds where normally WHO (dmft) is being used

^c^Based on the baseline assessment the participants were categorized into; Group I having sufficient oral health behaviours, Group II having moderate oral health behaviours and Group III having insufficient oral health behaviours and then the participants were followed for a 2-year period

^d^Oral hygiene measured by Oral Health index-Simplified (OHI-S)

^e^The CAST index scoring system is as follows: “0: sound”, “1: sealant”, “2: restoration”, “3: enamel lesions”, “4, 5: dentine lesions”, “6: pulp involvement”, “7: abscess/fistula”, “8: tooth loss”. If a situation did not match any codes from 0 to 8, a code 9 was assigned. The codes 0–2, 3, 4–5, 6–7, and 8 were considered as “healthy”, “pre-morbidity”, “morbidity”, “serious morbidity”, and “mortality”, respectively

**Table 5:** Statistically significant nutrition-related determinants contributing to dental caries

Determinants	Association: positive ( +), negative (−)^a^	Author (study design)	Country	Type of dentition	N	Age group (gender)	Study setting	Scoring system	Type/s of statistical analysis	Dental caries/scoring results
*Beverages*										
Soft drinks	+	Chedid et al. [49] (CS)	Lebanon	Primary	99	2–4 y (M, F)	Clinic	WHO (DFS score and bite wing radiograph)	Pearson’s	DCP = 74.7%
Soft drinks	+	Alhabdan et al. [60] (CS)	KSA	Primary	578	6–8 y (M)	School	WHO (dmft)	Adjusted Odds Ratios, Multivariate model logistic regression	DCCP: 83% dmft = 4.20 (SD 2.96)
Soft drinks	+	Hashim et al.^b^ [88] (CS)	UAE	Primary	1036	5–6 y (M, F)	School	WHO (dmft)	Adjusted Risk Ratio, Bivariate analysis	dmft = 4.5
Fruit juice- before bed	+	Al-Malik et al. [57] (CS)	KSA	Primary	987	2–5 y (M, F)	School	BASCD	Stepwise multiple logistic regression	DCP = 73% Rampant caries = 43% dmft = 4.8 dmfs = 12.7
Fruit juice-frequent consumption	+	Hashim et al.^b^ [88] (CS)	UAE	Primary	1036	5–6 y (M, F)	School	WHO	Risk Ratio, Bivariate analysis	dmft = 4.5
Citrus juice-frequent consumption (mixed dentition)	+	Abbass et al. [20] (CS)	Egypt	Primary Mixed Permanent	369	3–18 y (M, F)	Clinic	WHO (dmft, deft, DMFT)	Kruskal–Wallis, Spearman’s	DCP = 74% dmft = 3.23 (SD 4.07) deft = 4.21 (SD 3.21) DMFT = 1.04 (SD 1.56)
Fruit squash- frequent consumption	+	Huew et al. [51] (CS)	Libya	Permanent	791	12 y (M, F)	School	WHO (DMFT)	Multivariate stepwise regression	DCP = 57.8% DMFT = 1.68 DMFS = 2.38
Fruit squash- frequent consumption	+	Sayegh et al.^c^ [43] Sayegh et al.^c^ [45] (CS)	Jordan	Primary	1140	4–5 y (M, F)	School	WHO (dmft)	Multivariate analysis	DCP = 67% dmft > 4 in 31%
Fruit squash-frequent consumption	+	Al-Malik et al. [57] (CS)	KSA	Primary	987	2–5 y (M, F)	School	BASCD	Stepwise multiple logistic regression	DCP = 73% ECCP = 43% dmft = 4.8 dmfs = 12.7
Tea with sugar	+	Sayegh et al. [43] (CS)	Jordan	Primary	1140	4–5 y (M, F)	School	WHO (dmft)	Multivariate analysis	DCP = 67% dmft > 4 in 31%
Tea with sugar	+	Hashim et al.^b^ [88] (CS)	UAE	Primary	1036	5–6 y (M, F)	School	WHO (dmft)	Adjusted Risk Ratio Bivariate analysis	dmft = 4.5
Flavoured milk	+	Alhabdan et al. [60] (CS)	KSA	Primary	578	6–8 y (M)	School	WHO (dmft)	Adjusted Odds Ratios, Multivariate model logistic regression	DCCP = 83% dmft = 4.20 (SD 2.96)
Sweetened beverages^d^	+	Elamin et al. [89] (CS)	UAE	Primary	186	1.5–4 y (M, F)	School	WHO (dmft)	T-test, Pearson’s	DCP: 41% dmft = 1.7 (SD 2.81)
Sweetened beverages^d^	Unclear	Khadri et al. [90] (CS)	UAE	Permanent	803	11–17 y (M, F)	School	WHO (DMFT)	Multivariate regression	DCP = 75% DMFT = 3.19 (SD 2.9)
Sweetened beverages^d^	+	Ahmed et al. [41] (CS)	Iraq	Permanent	392	12 y (M, F)	School	WHO (DMFT)	ANOVA	DCP = 62% DMFT = 1.7
*Sugar rich food*										
Sugar containing foods^e^	+	Quadri et al. [67] (CS)	KSA	Primary Permanent	853	6–15 y (M, F)	School	WHO	Multi regression	DCP = 91.3%
Sugar containing foods^e^	+	Abbass et al. [20] (CS)	Egypt	Primary Mixed Permanent	369	3–18 y (M, F)	Clinic	WHO (dmft, deft, DMFT)	Kruskal–Wallis, Spearman’s	DCP = 74% dmft = 3.23 (SD 4.07) deft = 4.21 (SD 3.21) DMFT = 1.04 (SD 1.56)
Sugar containing foods^e^	+	Jaghasi et al. [72] (CS)	Syria	Not specified	504	6–12 y (M, F)	School	WHO	Logistic regression	DCP = 85%
Sugar containing foods^e^	+	Hashim et al.^a^ [88] (CS)	UAE	Primary	1036	5–6 y (M, F)	School	WHO (dmft)	Adjusted Risk Ratio, Bivariate analysis	dmft = 4.5
Sugar containing foods^e^- frequent consumption	+	Elamin et al. [89] (CS)	UAE	Primary	186	1.5–4 y (M, F)	School	WHO (dmft)	T-test, Pearson’s	DCP: 41% dmft = 1.7 (SD 2.81)
Sugar containing foods^e^- frequent consumption	+	Sayegh et al.^b^ [43] Sayegh et al.^c^ [45] (CS)	Jordan	Primary	1140	4–5 y (M, F)	School	WHO (dmft)	Multivariate analysis	DCP = 67% dmft > 4 in 31%
*Snacks and meal frequency*										
Sweet snacks^f^ and beverages	+	Kowash et al. [91] (CS)	UAE	Primary	540	4–6 y (M, F)	School	WHO (dmft)	Chi-Square	ECCP = 74.1% dmft = 3.01 SiC = 13.3
Sweet snacks^f^ and beverages	+	Kowash [92] (CS)	UAE	Primary	176	1.5–5 y (M, F)	Clinic	BASCD (dmft, dmfs)	Descriptive statistics	dmft = 10.9 dmfs = 32.1
Sweet snacks^f^ and beverages	+	Hashim et al. ^b^ [86] (CS)	UAE	Primary	1036	3–6 y (M, F)	School	WHO (ECC)	Logistic regression	Severe ECCP = 31.1%
Sweet snacks^f^-frequent consumption	+	Alhabdan et al. [60] (CS)	KSA	Primary	578	6–8 y (M)	School	WHO (dmft)	Adjusted odds ratios, Multivariate model logistic regression	DCCP = 83% dmft = 4.20 (SD 2.96)
Snacks-frequent consumption	+	Hashim et al.^b^ [87] (CS)	UAE	Primary	1036	5–6 y (M, F)	School	WHO (dmft)	Adjusted Risk Ratio, Bivariate analysis	dmft = 4.5
Snacks	+	Chedid et al. [49] (CS)	Lebanon	Primary	99	2–4 y (M, F)	Clinic	WHO (DFS score and bite wing radiographs)	Pearson’s	DCP = 74.7%
Milk-as snack	−	Chedid et al. [49] (CS)	Lebanon	Primary	99	2–4 y (M, F)	Clinic	WHO (DFS score and bite/wing radiograph)	Pearson’s	DCP = 74.7%
Main meal consumption	Unclear	Khadri et al. [90] (CS)	UAE	Permanent	803	11–17 y (M, F)	School	WHO (DMFT)	Multivariate regression	DCP = 75% DMFT = 3.19 (SD 2.9)
Eating frequently (> 5times daily)	+	Hashim et al.^a^ [88] (CS)	UAE	Primary	1036	5–6 y (M, F)	School	WHO (dmft)	Adjusted Risk Ratio, Bivariate analysis	dmft = 4.5
*Other eating related factors*										
No fruit consumption-	−	Alhabdan et al. [60] (CS)	KSA	Primary	578	6–8 y (M)	School	WHO (dmft)	Adjusted Odds Ratios Multivariate model logistic regression	DCCP = 83% dmft 4.20 (SD 2.96)
Sweet taste perception	+	Ashi et al. [63] (CS)	KSA^g^	Permanent	225	15–15 y (M, F)	School	ICDAS, (DMFS)	One-way ANOVA LSD	DMFS = 2.99
Low dietary score^h^	+	Al-Otaibi et al. [93] (CS)	Yemen	Not specified	400	12 y (M, F)	School	WHO (DMFT)	Multivariate logistic regression,	DCP = 90.2% DMFT = 2.22
Low nutrient food^i^-frequent consumption	+	İnan-Eroğlu et al. [78] (CS)	Turkey	Primary	395	36–71 m (M, F)	School	WHO (dmft, dmfs)	Mann–Whitney, Kruskal–Wallis	dmft = 4.7 dmfs = 8.0
Dairy products-low consumption	+	Jaghasi et al. [72] (CS)	Syria	Not specified	504	6–12 y (M, F)	School	WHO	Logistic regression	DCP = 85%
Cod liver intake	−	Bener et al. [55] (CS)	Qatar	Permanent	1284	6–15 y (M, F)	Clinic	WHO (DMFT)	Multivariate analysis	DCP = 73% DMFT = 4.5
Nutritious food^j^-frequent consumption	−	Abbass et al. [20] (CS)	Egypt	Primary Mixed Permanent	369	3–18 y (M, F)	Clinic	WHO (dmft, deft, DMFT)	Kruskal–Wallis, Spearman’s	DCP = 74% dmft = 3.23 (SD 4.07) deft = 4.21 (SD 3.21) DMFT = 1.04 (SD 1.56)
*Infant feeding practices*										
Feeding type^k^	+	Abu Hamila [21] (CS)	Egypt	Primary	560	1–3.5 y (M, F)	Clinic	WHO (dmft)	Chi-Square	ECCP = 69.6% dmft range = 2.1–7.6
Breastfeeding-Long duration	+	Sayegh et al.^c^ [44] Sayegh et al.^c^ [45] (CS)	Jordan	Primary	1140	4–5 y (M, F)	School	WHO (dmft)	Multivariate analysis	DCP = 67% dmft > 4 in 31%
Breastfeeding -On demand feeding	+	Sayegh et al.^c^ [44] Sayegh et al.^c^ [45] (CS)	Jordan	Primary	1140	4–5 y (M, F)	School	WHO (dmft)	Multivariate analysis	DCP = 67% dmft > 4 in 31%
Formula feeding	+	Alhabdan et al. [60] (CS)	KSA	Primary	578	6–8 y (M)	School	WHO (dmft)	Adjusted Odds Ratios, Multivariate model logistic regression	DCCP = 83% dmft = 4.20 (SD 2.96)
Formula feeding	+	Bener et al. [55] (CS)	Qatar	Permanent	1284	6–15 y (M, F)	Clinic	WHO (DMFT)	Multivariate analysis	DCP = 73% DMFT = 4.5
Formula feeding	+	Qadri et al. [73] (CS)	Syria	Primary	400	3–5 y (M, F)	School	ECC WHO (dmft, dmfs)	Logistic regression	ECCP = 48% DCP = 70% dmft = 4.25 (SD 4.24)
Night feeding -bottle	+	Mohebbi [33] (CS)	Iran	Primary	504	1–3 y (M, F)	Clinic	WHO	T-test, Chi-Square, ANOVA, Logistic regression	DCP = 3–26% depending on age
Night feeding -bottle	+	Ozer et al. [83] (CS)	Turkey	Primary	226	3–6 y (M, F)	School	WHO (dmft) AAPD (ECC)	Bivariate analysis	ECCP = 46.9% dmft = 2.87
Night feeding	+	Kabil & Eltawil, 2016 [18] (CS)	Egypt	Primary	140	2–4 y (M, F)	Clinic	WHO (DMFT)	Logistic regression	DMFT = 9.96
Night feeding	+	Kabil & Eltawil [19] (CS)	Egypt	Primary	108	2–4 y (M, F)	Clinic	WHO (ECC)	Logistic regression	ECCP = 57% (2–3 y), 73% (3–4 y)
Bottle feeding-on demand	+	Sayegh et al.^c^ [44] Sayegh et al.^c^ [45] (CS)	Jordan	Primary	1140	4–5 y (M, F)	School	WHO (dmft)	Multivariate analysis	DCP = 67% dmft > 4 in 31%
Sleep with bottle	+	Alhabdan et al. [60] (CS)	KSA	Primary	578	6–8 y (M)	School	WHO (dmft)	Adjusted Odds Ratios, Multivariate model logistic regression	DCCP = 83% dmft = 4.20 (SD 2.96)
Sleep next to mother	+	Sayegh et al.^c^ [44] Sayegh et al.^c^ [45] (CS)	Jordan	Primary	1140	4–5 y (M, F)	School	WHO (dmft)	Multivariate analysis	DCP = 67% dmft > 4 in 31%
Dummy use	+	Sayegh et al.^c^ [44] Sayegh et al.^c^ [45] (CS)	Jordan	Primary	1140	4–5 y (M, F)	School	WHO (dmft)	Multivariate analysis	DCP = 67% dmft > 4 in 31%
Dummy-sweetened	+	Al-Malik et al. [57] (CS)	KSA	Primary	987	2–5 y (M, F)	School	BASCD (dmft, dmfs))	Logistic regression	DCP = 73% ECCP = 43% dmft = 4.8 dmfs = 12.67
Shared spoons between mother and child^l^	+	Cogulu et al. [76] (L-24 m)	Turkey	Primary	92	15–35 m (M, F)	Clinic	WHO (dft, dfs)	Logistic regression	Final DCP = 45% Final dft = 1.0 Final dfs = 1.8

*AAPD* American Association Paediatric Dentistry, *BASCD* British Association for the Study of Community Dentistry, *CS* Cross-sectional, *CC* Case control, *DCP* Dental caries prevalence, *deft* decayed, extracted due to caries and filled primary teeth, *dfs* decayed, filled surfaces in primary teeth, *dft* decayed, filled primary teeth, *dmfs* decayed, missing and filled surfaces in primary teeth; *DMFS* decayed, missing and filled surfaces in permanent teeth, *dmft* decayed, missing, filled primary teeth, *DMFT* decayed, missing, filled permanent teeth, *ECC* Early childhood caries, *ECCP* Early childhood caries prevalence, *F* Female, *ICADS* The international caries Detection and Assessment System, *L* Longitudinal, *KSA* Kingdom of Saudi Arabia, *m* months *M* Male, *WHO* World Health Organisation, *SiC* Significant caries index, *SD* Standard deviation, *y* years

^a^Association: Positive ( +), negative (−) refers to this factor being either a statistically significant risk factor for caries (positive, +) or to this factor being statistically significant protective against caries (negative, −). In some studies it could not be determined whether a factor was positively or negatively associated with caries and in these cases the relation is described as unclear

^b^Hashim et al. 2006, Hashim et al. 2009, Hashim et al. 2011 and Hashim et al. 2013 seem to be based on the same study population but reporting different results

^c^Sayegh et al. 2002 and Sayegh et al. 2005 seem to be based on the same study population and the results mentioned in this table, have been reported in both articles

^d^Sweetend beverages refer to the consumption of various sweet beverages like soft drinks, fruit squashes, tea with sugar, flavoured milk, etc.

^e^Sugar rich food may include consumption of all/and mix of items like candy, chocolates, dates, ice-cream, cakes, muffins, etc.

^f^Sweet snacks include various food items with high sugar content

^g^KSA was part of this multinational study which also included Italy and Mexico. Only the results for KSA are presented in this table

^h^The dietary score was based on a few questions related to the consumption of cariogenic food and eating patterns with yes/no answer options

^i^Assessed by the Healthy Eating Index (HEI) 2010 and the Mediterranean Diet Quality Index for children and adolescents (KIDMED)

^j^Nutritious food refers to a frequent consumption of high nutrient food like fruits, vegetables, beans, milk, eggs etc.

^k^The feeding type had an impact on the caries prevalence as follows: 75.39% of breastfeed children, 70.39% of the formula fed, 68.67% of those who were weaned and 55% of those who got a mix of breast milk and formula had dental caries respectively

^l^During the baseline sampling mothers reported that they put their child’s spoon into their own mouth while feeding their child

### Determinants related to child characteristics

Table 2 describes the statistically significant determinants contributing to dental caries that were related to children’s sex, age and weight status. Increased age was associated with a higher risk of caries in 19 studies across eight countries [20, 21, 23, 26, 28, 31, 33, 37, 43, 45, 46, 55, 57, 69, 73, 77, 78, 80, 82]. Nine studies reported a higher risk of dental caries in males [18–21, 27, 35, 36, 47, 85], while females were reported to have a higher caries risk in six studies [26, 30, 31, 50, 55, 65]. Weight status was significantly associated with caries in nine studies, of which four studies reported positive associations between high BMI/overweight and caries [25, 30, 55, 56] and two studies reported an inverse association between BMI and dental caries [47, 59]. Two studies showed a positive association between low BMI/weight and caries [68, 79], and one study reported that normal weight children had a lower caries prevalence than either over- or underweight children [64] (Table 2).

### Determinants related to family background characteristics

Table 3 describes the statistically significant determinants related to family background, such as socioeconomic, sociodemographic, geographical location, school type (private or public), and parents’ education level, as potential risk factors contributing to dental caries. A total of 20 studies found negative associations with maternal education (13 studies) [21, 26, 29, 31, 37, 52, 57, 58, 60, 67, 81, 83, 89], paternal education (3 studies) [24, 27, 50], or education of both parents combined (4 studies) [20, 38, 40, 42] (Table 3).

Parents’ employment status was found to be either positively or negatively associated with caries in seven studies [21, 22, 32, 37, 38, 62, 80]. Although there was no coherent measurement of socioeconomic status between the reviewed studies, overall socioeconomic status (SES), income, affluence or access to dental insurance were found to have a negative association with dental caries in seven studies, whereas Bener et al. found a positive association between household income and dental caries in Qatar [55]. In addition, significant associations were found between family size, order and numbers of siblings, rural or urban residency, nationality and school type in various studies (Table 3).

### Determinants related to oral hygiene

In the reviewed studies, oral hygiene and oral practices were assessed directly using plaque or oral hygiene indices or indirectly using self-reports by parents/guardians or participants. Table 4 illustrates statistically significant oral hygiene-related determinants contributing to dental caries. In 11 studies, an association between the frequency of tooth brushing and dental experience was found with reduced dental caries prevalence among those who frequently brushed their teeth and vice versa [20, 22, 37, 40, 55, 60, 66, 81, 84, 89, 91]. Some studies reported an association between parental-related factors such as supervision of tooth brushing (mainly in primary dentition), parental knowledge about oral hygiene, or parental caries status and the caries experience in their children (Table 4).

### Determinants related to infant feeding and eating habits

Table 5 presents the statistically significant determinants/risk factors related to infant feeding and eating habits contributing to dental caries. Infant feeding practices such as breastfeeding, bottle feeding and mixed feeding were all positively associated with dental caries in different studies. Furthermore, four studies found a positive association between night feeding and caries [18, 19, 34, 83]. Other factors, such as bottle feeding on demand, sleeping with the bottle, sleeping next to the mother, using a (sweetened) dummy, or sharing a spoon with the mother, were also positively associated with caries (Table 5).

The consumption of sweet beverages such as soft drinks (3 studies) [49, 60, 88], fruit juices (3 studies) [20, 57, 88], fruit squashes (3 studies) [43, 51, 57], tea with sugar (2 studies) [43, 88], flavoured milk (1 study) [60] and sweet beverages in general (2 studies) [41, 89] was positively associated with caries (Table 5). Sugar-containing foods such as cakes, muffins, chocolates, sweets and similar foods were also positively associated with caries in six studies [20, 43, 67, 72, 88, 89]. Higher frequency and/or sweet food snacking/eating was positively associated with caries in six studies [49, 60, 86, 87, 91, 92], whereas one Lebanese study found that drinking milk as a snack was inversely associated with caries [49]. Other factors, such as cod liver intake, frequent consumption of nutritious food and no fruit consumption, were found to be negatively associated with caries, whereas sweet taste perception, low intake of nutrient-dense food and low dairy product consumption were positively associated with dental caries (Table 5).

## Discussion

The purpose of this review study was to identify, gather, assess and summarize evidence from scientific studies to address sociobehavioural/cultural and socioeconomic determinants of dental caries among children residing in the MENA region. A structured approach was used to identify 77 relevant studies from 14 countries (Egypt, Iran, Iraq, Jordan, Kuwait, Lebanon, Libya, Palestine, Qatar, Saudi Arabia, Syria, Turkey, UAE, and Yemen), whereas no relevant studies were found from Algeria, Bahrain, Morocco, Oman and Tunisia, highlighting a knowledge gap about children’s dental status in these specific countries. This study showed a high caries prevalence in many studies regardless of age group or publication date, indicating a worsening dental health status in the MENA region compared to previous reports [95]. The most commonly reported socioeconomic/demographic and behavioural determinants of dental caries in children reported in this review included low parental education level, low household income, frequent consumption of sugars and/or poor dietary habits and poor oral habits, including tooth brushing, dental visits and parental engagement or knowledge on oral hygiene.

### Dental caries prevalence and trends

Over half of the reviewed articles originated from Iran (18 studies) [22–39], Saudi Arabia (14 studies) [56–69], Turkey (11 studies) [74–84], and the UAE (8 studies) [85–92], with the vast majority being cross-sectional, presenting a snap shot of the regional prevalence of dental caries rather than the development over time. However, based on the available literature from Iran, Saudi Arabia and Turkey, some dental caries patterns and/or trends could be observed. In 2004 and 2006, the dental caries prevalence among Iranian children below the age of 6 years was reported to be 17.2% and 3–26%, respectively [23, 33]. In 2011, Amanlou et al. reported a prevalence of 49.3%, whereas studies published in 2017 or later showed a prevalence of 69.9% and 87%, respectively, indicating a clear trend towards an increased prevalence of dental caries among young children in Iran over the past 15 years [22, 29, 37]. Similar to a previous review study, an increased prevalence of caries has been shown over the past few decades in Saudi Arabia [96]*.* In this investigation, the four studies published in 2008–2018 reported the dental caries prevalence to be 49–91.3% in different locations of Saudi Arabia [61, 62, 64, 67]. Likewise, in Turkey, high prevalence was also observed among children below the age of 6 years, where five out of the six studies published in 2003–2011 showed that at least three-quarters of the children had dental caries [76, 79–82]. Similar to the findings in Saudi Arabia and Turkey, studies from many other MENA countries also reported a high prevalence of dental caries, indicating a concerning development regarding dental status in the region. Sheiham and Williams reported an increased prevalence of dental caries in many African and Middle Eastern countries, supporting these findings [97].

### Age and gender as determinants for dental caries

Increased age was identified as an independent risk factor for dental caries in several studies, probably reflecting the cumulative effect of the disease, which is on par with the literature [14, 98]. Although females may be expected to exhibit a higher caries rate due to earlier tooth eruption, and thus longer exposure to cariogenic processes, variations in the associations between sex and dental caries were found in this study. Female sex was associated with a higher risk in six studies, whereas males were at a higher risk in eight of the studies. Others have attributed sex variations to differences in dietary and oral hygiene behaviours or utilization of oral health care [99, 100].

### Sociodemographic determinants for dental caries

The role of parental variables that are directly associated with children's oral health, including sociodemographic characteristics, oral health behaviours, access to health services and other attributes, is evident in this study. In a recent study, this was validated through a conceptual model [101]. SES is generally measured by indicators of human capital, such as income, education, urban/rural living, and occupational nature, which offer advantages or disadvantages to individuals and families. In line with findings from other regions and despite the differences in measuring SES in the reviewed articles, socioeconomic factors were shown to have a significant impact on dental caries [14]. It was primarily mothers’ level of education, but also other factors, such as parental occupation, unemployment, low-skilled occupation, low income, overall SES and school type, that were identified as determinants of caries (Table 2).

### Dietary determinants for dental caries

Most dietary determinants for caries were related to sugar intake: consumption, amount, frequency or timing of sweet beverages, snacks and/or food. The current findings in establishing sugar intake and SES factors as key determinants of dental caries in the region are consistent with those of studies in several other countries that have demonstrated socioeconomic gradients in sugar consumption and may accordingly prompt dietary recommendations in limiting added sugar intake and targeting SES disadvantaged groups in the region [102–104]. Moreover, other determinants were identified, such as a lack of an overall healthy diet or intake of certain nutritious foods, which again emphasizes the importance of promoting healthy eating habits and the need for dietary guidelines.

Regarding infant feeding practices, the findings in this study were inconclusive, indicating that both bottle feeding and breastfeeding were associated with higher caries prevalence in different studies [21, 44, 55, 60]. These findings are in contrast with those in a systematic review and meta-analysis that concluded that breastfeeding seems to be protective against dental caries when compared to bottle feeding [105].

### Oral hygiene determinants for dental caries

Tooth brushing as a determinant for caries was a distinct finding in this study; a reduced dental caries experience could be found among those who frequently brush their teeth and vice versa, and this was more apparent in the young age groups with primary dentition [20, 22, 37, 40, 55, 60, 66, 81, 89, 91]. Additional determinants related to tooth brushing included age of brushing initiation, frequency, adult supervision and the presence of visible plaque. These factors are all interrelated factors that could potentially also be linked to SES [106, 107].

### Methodological considerations

In this review, the associations between determinants and dental caries were mainly projected from cross-sectional studies. These methodological choices, i.e., the study design (cross-sectional), sampling procedures (e.g., non–population based, convenience sampling), assessment setting and/or outcome measures may be an expected consequence of the relatively immature research infrastructure, limited resources in some of the MENA countries or may be related to social or political turmoil that some countries have experienced [41, 108]. Although cross-sectional studies may be a feasible option in such circumstances, they only provide a snapshot of risk factors that are associated with the outcome, but causal pathways cannot be determined since the exposure and outcome are measured simultaneously [109]. On the other hand, case–control and longitudinal studies offer greater scientific evidence through better control of possible methodological biases and data analysis, and over time, these types of studies will be needed to further develop and strengthen the research landscape [108]. The aforementioned imbalance in research output between countries hinders the establishment of a comprehensive dental caries profile of the MENA region. This imposes the need to increase dental caries research output in some countries and to devote more rigorous, unique (not repetitive), up-to-date and representative research from others. These steps can strengthen the ability to comprehensively assess trends and determinants of dental caries in the region, allow for cross-country comparisons and identify regional variations in the future.

### Strengths and limitations

The strengths of this study include the systematic approach employed in assessing the articles published during a period of 20 years focusing on children and young adults. Furthermore, this study focused mainly on modifiable determinants in a region with a young population, which can guide informed dental public health actions and thereby decrease health inequities. The results in this study were reported without assessing the strength/power or the quality of either the study design, sampling procedures or the statistical analysis of the included articles, which can be seen as limitations. Furthermore, the methodological heterogeneity (study design, age group, exposure, outcome measurements, covariates, statistical analyses) among the studies included in this article may have influenced the interpretation of the results; hence, these findings need to be confirmed or rejected by future studies. However, drawing the comprehensive landscape of the disease and its determinants offers an outlook in a relatively understudied region which is a prerequisite for designing follow-up studies. Future studies may focus on appraisal and quality assessment of the reviewed studies, using tools such as those suggested by Migliavaca et al. for prevalence studies [110].

## Conclusions

To conclude, **t**he prevalence of dental caries among children and young adults in the MENA region was high. Despite heterogeneity in the study designs and assessment methods of dental caries, the main determinants of dental caries were found to include age, sex, mother’s education, overall socioeconomic status, tooth brushing frequency, parents’ oral habits/knowledge and sugar consumption. The high dental caries prevalence imposes the need to address the prevailing modifiable sociobehavioural and socioeconomic determinants by translating them into effective oral health prevention policies and programmes. Moreover, a special emphasis on strengthening regional oral health research would further enhance the knowledge and understanding of a major public health problem in the region.

## Data Availability

The dataset generated and analysed for the current study is not publicly available, but data are available from the corresponding author on reasonable request.

## Abbreviations

AAPD: American Association Paediatric Dentistry
ASTDD: Association of State and Territorial Dental Directors
BASCD: British Association for the Study of Community Dentistry
dmft/DMFT: Decayed, missing, and filled (primary/permanent) teeth scores
EMRO: Eastern Mediterranean Region
ICADS: International Caries Detection and Assessment System
MDGs: Millennium Development Goals
MENA: Middle East and North Africa
SES: Socioeconomic status
SDG: Sustainable Development Goals
WHO: World Health Organization
